# Cryptotanshinone chemosensitivity potentiation by TW-37 in human oral cancer cell lines by targeting STAT3–Mcl-1 signaling

**DOI:** 10.1186/s12935-020-01495-2

**Published:** 2020-08-26

**Authors:** In-Hyoung Yang, Seung-Hyun Hong, Minjung Jung, Chi-Hyun Ahn, Hye-Jung Yoon, Seong Doo Hong, Sung-Dae Cho, Ji-Ae Shin

**Affiliations:** 1 Cancer Center, Texas Tech University Health Sciences Center, Lubbock, TX 79430 USA; 2 Department of Pediatrics, Texas Tech University Health Sciences Center, Lubbock, TX 79430 USA; 3 Department of Oral Pathology, School of Dentistry and Dental Research Institute, Seoul National University, Seoul, 03080 Republic of Korea

**Keywords:** TW-37, Cryptotanshinone, Chemosensitivity, STAT3, Mcl-1, Oral cancer, Apoptosis

## Abstract

**Background:**

Despite being one of the leading cancer types in the world, the diagnosis of oral cancer and its suitable therapeutic options remain limited. This study aims to investigate the single and chemosensitizing effects of TW-37, a BH3 mimetic in oral cancer, on human oral cancer cell lines.

**Methods:**

We assessed the single and chemosensitizing effects of TW-37 in vitro using trypan blue exclusion assay, Western blotting, DAPI staining, Annexin V–FITC/PI double staining, and quantitative real-time PCR. Mcl-1 overexpression models were established by transforming vector and transient transfection was performed to test for apoptosis

**Results:**

TW-37 enhanced the cytotoxicity of human oral cancer cell lines by inducing caspase-dependent apoptosis, which correlates with the reduction of the myeloid cell leukemia-1 (Mcl-1) expression via transcriptional and post-translational regulation. The ectopic expression of Mcl-1 partially attenuated the apoptosis-inducing capacity of TW-37 in human oral cancer cell lines. Besides, TW-37 decreased the phosphorylation of signal transducer and activator of transcription 3 (STAT3) at Tyr^705^ and nuclear translocation in human oral cancer cell lines at the early time points. Furthermore, TW-37 potentiated chemosusceptibility of cryptotanshinone in human oral cancer cell lines by suppressing STAT3–Mcl-1 signaling compared with either TW-37 or cryptotanshinone alone, resulting in potent apoptosis.

**Conclusions:**

This study not only unravels the single and chemosensitizing effects of TW-37 for treatment of human oral cancer but also highlights the likelihood of TW-37 as a good therapeutic strategy to enhance the prognosis of patients with oral cancer in the future.

## Background

Globally, head and neck squamous cell carcinoma (HNSCC) is one of the leading cancer types, of which oral squamous cell carcinoma constitutes > 90% of cases [1]. Despite witnessing a decline in the incidence of some cancer types, the incidence and mortality of oral cancer have steadily increased in both males and females [2]. The standard therapeutic options for managing patients with oral cancer include surgery, radiotherapy, and chemotherapy, which depend on the type, location, and stage of cancer at diagnosis. Despite significant advancements in the approaches of oral cancer treatment over the past decades, effective diagnosis and suitable therapeutic options of oral cancer remain limited, thereby triggering low prognosis of patients [3]. Hence, finding suitable therapeutic options for the treatment of patients with oral cancer is of utmost significance to enhance the therapeutic outcome.

In cancer therapy, targeting anti-apoptotic Bcl-2 family proteins is an attractive approach because of their oncogenic potential. The “BH3 mimetics” were designed to mimic the BH3 domain of pro-apoptotic Bcl-2 proteins and to antagonize anti-apoptotic Bcl-2 family proteins, resulting in apoptosis [4]. Of these, TW-37 is an analog of gossypol, a plant-derived polyphenolic aldehyde, which can bind to Bcl-2, Bcl-xL, and myeloid cell leukemia-1 (Mcl-1) in the nanomolar range [5, 6]. A growing number of studies have demonstrated the therapeutic potential of TW-37 against various types of cancer by inducing S-phase cell cycle arrest or apoptosis in vitro and in vivo [7–9]. In HNSCC, TW-37 has also been reported to suppress tumor angiogenesis and sensitize the antitumor effect of cisplatin [10]. Recently, our team demonstrated that TW-37 functions as a potential apoptosis-inducing agent for the treatment of oral cancer by reducing heme oxygenase-1 and Bcl-2 [11, 12]. Furthermore, in combination with conventional chemotherapeutic drugs like cisplatin and 5-fluorouracil, TW-37 was sufficient in potentiating the chemosensitivity of tumors by inducing apoptosis [13, 14]. Hence, therapeutic approaches by TW-37 alone or combined with chemotherapeutic drugs are crucial to augment the survival of patients with oral cancer.

Based on the related literature [15], this study hypothesizes that the transcriptional regulation of Mcl-1 in human oral cancer cell lines upon TW-37 treatment could correlate with the signal transducer and activator of transcription (STAT) family members, including STAT3 (H1). In addition, we hypothesize that the combination of TW-37 and cryptotanshinone at low concentration ranges could potently suppress the Mcl-1 expression by inhibiting the STAT3 activation (H2). Hence, this study aims to explore whether TW-37 could be a valuable therapeutic option as monotherapy in human oral cancer, as well as combination therapy with cryptotanshinone, by targeting STAT3–Mcl-1 signaling.

## Methods

### Cell culture and chemical preparation

HSC-3, Ca9.22, and HSC-4 cell lines are obtained from Hokkaido University (Hokkaido, Japan), and the HN22 cell line was provided by Dankook University (Cheonan, Republic of Korea). All cell lines were maintained in DMEM/F12 medium (WELGENE, Gyeongsan, Republic of Korea) supplemented with 10% fetal bovine serum and 1% antibiotics (penicillin/streptomycin) at 37 °C in a 5% CO_2_ incubator. In addition, TW-37 was purchased from ApexBio (Houston, TX, USA), whereas cryptotanshinone and cycloheximide (CHX) were purchased from Sigma-Aldrich (St Louis, MO, USA). ABT-737 and Stattic were supplied from Selleckchem (Houston, TX, USA), Z-VAD-FMK was purchased from R&D Systems (Minneapolis, MN, USA), and MG132 was provided from Santa Cruz Biotechnology (Santa Cruz, CA, USA). All chemicals were dissolved in dimethyl sulfoxide and stored at −20 °C.

### Trypan blue exclusion assay

**Fig. 1:**
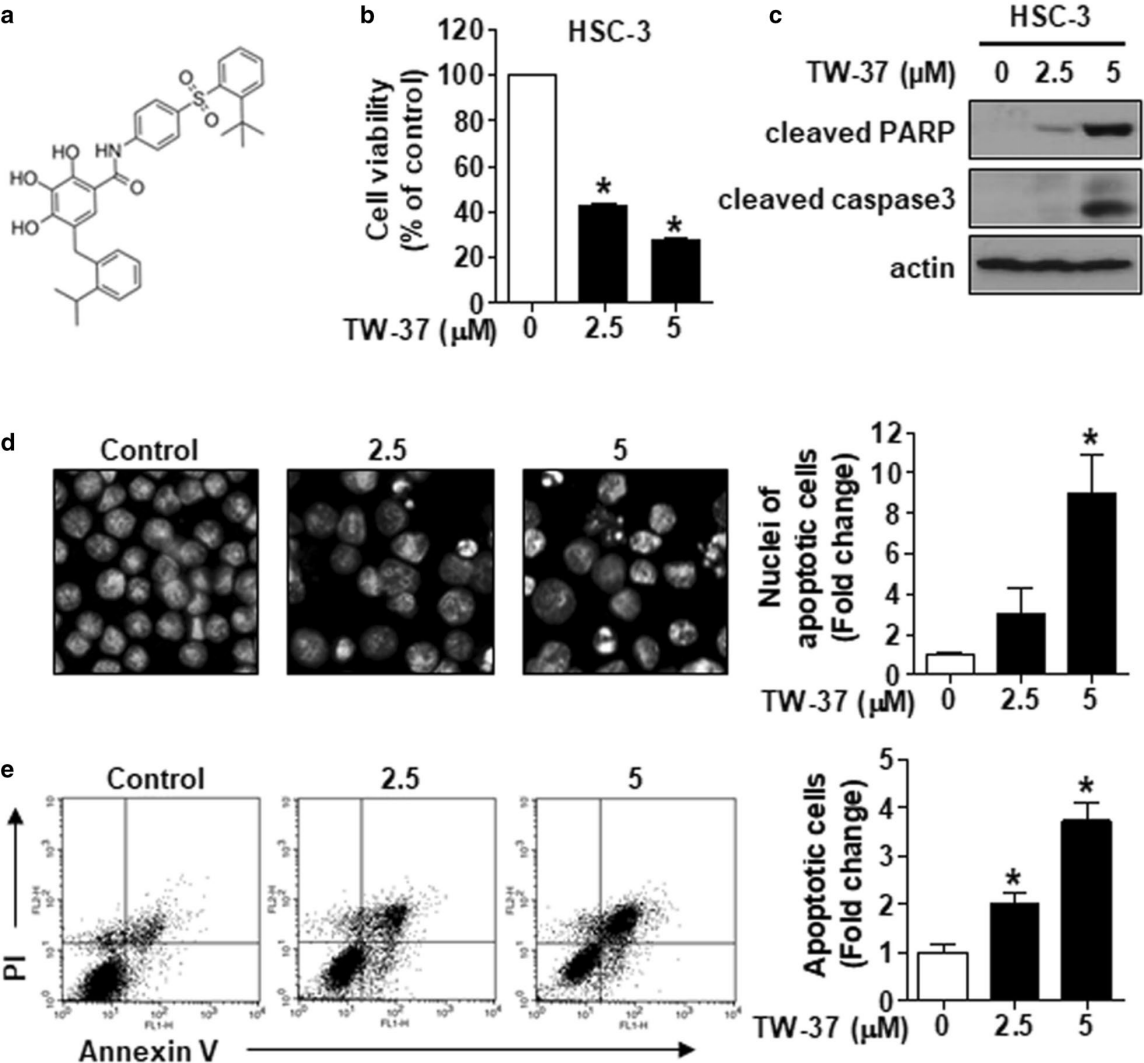
TW-37 augments the cytotoxicity of human oral cancer cell lines by triggering apoptosis. **a** The chemical structure of TW-37. **b** HSC-3 cell lines were treated with 2.5- and 5-μM TW-37 for 48 h, following which the cell viability was measured using the trypan blue exclusion assay. **c** Cleavage of both PARP and caspase-3 was detected by Western blotting. Actin was used as a loading control. **d** HSC-3 cell lines were stained with DAPI and visualized by fluorescence microscopy (magnification, ×400). **e** FACS analysis of annexin V/PI staining. All bar graphs represent mean ± SD of the three independent experiments. *P < 0.05

We seeded all cell lines in six-well plates and treated those with TW-37. Figure 1a shows the structure of TW-37. Then, 48 h after the TW-37 treatment, all cell lines were stained with 0.4% trypan blue solution (Gibco, Paisley, UK), and viable cell lines were counted using a hemocytometer.

### Western blotting

All cell lines were extracted with 1X RIPA lysis buffer (EMD Millipore, Billerica, CA, USA) using phosphatase inhibitor tablets (Thermo Scientific Inc., Rockford, IL, USA) and protease inhibitor cocktails (Roche, Mannheim, Germany). We then performed protein quantification using a DC protein assay kit (Bio-Rad Laboratories, Madison, WI, USA). After normalization, protein lysates containing nearly 20–50 μg of protein were boiled with a protein sample buffer at 95 °C for 5 min and separated on SDS–PAGE. Next, the proteins were transferred to Immun-Blot PVDF membranes after the electrophoresis and blocked with 5% skimmed milk for 1 h at room temperature (RT). The membranes were incubated with the indicated primary antibodies overnight at 4 °C and were maintained with corresponding horseradish peroxidase (HRP)–conjugated secondary antibodies for 2 h at RT. The antibodies for all the target proteins have been listed in Table 1. The bands were then immune-reactivated with an ECL solution (Santa Cruz Biotechnology) and visualized using ImageQuant LAS 500 (GE Healthcare Life Sciences, Piscataway, NJ, USA) or an X-ray film.

**Table 1:** List of antibodies and dilutions used in Western blot

Antibody	Manufacturer	Catalog number	Dilution
Cleaved PARP	Cell Signaling Technology	9541	1:3000
Cleaved caspase 3	Cell Signaling Technology	9664	1:3000
Actin	Santa Cruz Biotechnology	sc-47778	1:5000
Mcl-1	Cell Signaling Technology	5453	1:3000
Bcl-2	Santa Cruz Biotechnology	sc-7382	1:3000
Bcl-xL	Cell Signaling Technology	2764	1:3000
p-STAT3(Y705)	Cell Signaling Technology	9145	1:3000
p-STAT3(S727)	Cell Signaling Technology	9134	1:3000
STAT3	Cell Signaling Technology	4904	1:3000
p-STAT5(Y694)	Cell Signaling Technology	4322	1:3000
STAT5	Cell Signaling Technology	4904	1:3000
α-Tubulin	Santa Cruz Biotechnology	sc-5286	1:3000
Lamin B	Santa Cruz Biotechnology	sc-6217	1:3000
Goat IgG antibody(HRP)	GeneTex	GTX232040-01	1:5000
Mouse IgG antibody(HRP)	GeneTex	GTX213111-01	1:2000
Rabbit IgG antibody(HRP)	GeneTex	GTX213110-01	1:5000

### DAPI staining

After the TW-37 treatment, all cell lines were fixed overnight with 100% ethanol at −20 °C and 100% methanol at RT for 10 min. The fixed cell lines were then deposited on slides and stained with DAPI solution (5 μg/mL). Furthermore, we observed morphological changes of nuclei in apoptotic cell lines using a fluorescence microscope (Leica DMi8; Leica Microsystems GmbH, Wetzlar, Germany).

### Annexin V–FITC/PI double staining

We measured the induction of apoptosis using the FITC Annexin V Apoptosis Detection Kit (BD Pharmingen, San Jose, CA, USA) according to the manufacturer’s protocol. Briefly, all floating and adherent cell lines were collected, washed two times with PBS, and pelleted by centrifugation. After that, cell lines were resuspended in annexin V binding buffer containing 3-μL annexin V–FITC and 1-μL PI and incubated for 15 min at RT in the dark. Subsequently, we transferred cell lines to a FACS tube and analyzed by flow cytometry using a FACSCalibur and counted 10,000 events per sample.

### Quantitative real-time PCR

Using TRIzol Reagent (Life Technologies, Carlsbad, CA, USA), the total RNA was extracted. Afterward, 1 μg of RNA was reverse transcribed using an AMPIGENE cDNA Synthesis Kit (Enzo Life Sciences, Inc., NY, USA), and the resultant cDNA was subjected to PCR using AMPIGENE qPCR Green Mix Hi-Rox (Enzo Life Sciences). We performed real-time PCR using the StepOnePlus Real-Time PCR System (Applied Biosystems, CA, USA). In this study, the PCR conditions for all genes were as follows: 95 °C for 2 min, followed by 40 cycles of 95 °C for 10 s and 60 °C for 30 s. The relative amount of *Mcl-1* gene was normalized to the amount of *GAPDH* and was evaluated using the 2^–ΔΔCt^ method. The qPCR primers were as follows: *Mcl-1* sense, 5′-GTA TCA CAG ACG TTC TCG TAA GG-3′; *Mcl-1* antisense, 5′-CCA CCT TCT AGG TCC TCT ACA T-3′; *GAPDH* sense, 5′-GTG GTC TCC TCT GAC TTC AAC-3′; and *GAPDH* antisense, 5′-CCT GTT GCT GTA GCC AAA TTC-3′.

### Plasmid construction and transient transfection

We amplified the open reading frame of human *Mcl-1* (NM_021960) gene from cDNA that was synthesized in HSC-3 cell lines using gene-specific primers (primer sequence: *Mcl-1* sense, 5′-GAA TTC ATG TTT GGC CTC AAA AGA-3′, with an included *Eco*RI site; *Mcl-1* antisense, 5′-GAA TTC CTA TCT TAT TAG ATA TGC-3′, with an included *Eco*RI site), followed by cloning into a pGEM® T-easy vector (Promega, Madison, WI, USA). The *Mcl-1* was then established by sequence analysis. Finally, we cloned the *Mcl-1* gene into the multi-cloning site of the pcDNA3.1 (+) vector (Invitrogen, San Diego, CA, USA). For the overexpression vector transfection, we transfected cell lines with vector constructs (pcDNA3.1 or pcDNA3.1-Mcl-1) using Lipofectamine 3000 transfection reagent (Life Technologies) according to the manufacturer’s protocol.

### Statistical analysis

We compared the control and the treated groups through Student’s *t*-test (two tailed) using SPSS 22 (SPSS, Chicago, IL, USA). In this study, *P* < 0.05 was considered statistically significant.

## Results

### TW-37 represses cell viability and induces apoptosis in human oral cancer cell lines

We first performed a concentration–response test using the trypan blue exclusion assay to assess the potential cytotoxic effect of TW-37 on human oral cancer cell lines. TW-37 exhibited a concentration-dependent cytotoxic effect on HSC-3 cell lines (Fig. 1b). In addition, similar cytotoxicity was observed in the three human oral cancer cell lines, including Ca9.22, HSC-4, and HN22 (Additional file 1: Figure S1a). Furthermore, we analyzed the levels of cleaved caspase-3 and cleaved PARP using Western blotting to determine whether the cytotoxic effect of TW-37 was because of the induction of apoptosis. TW-37 induced apoptosis in four human oral cancer cell lines (Fig. 1c; Additional file 1: Figure S1b). Likewise, morphological changes in nuclei by fluorescence-based analysis exhibited TW-37-induced apoptosis in four human oral cancer cell lines, as evidenced by chromatin condensation or DNA fragmentation (Fig. 1d; Additional file 1: Figure S1c). Next, we examined the apoptotic effect of TW-37 using flow cytometric analysis. The rate of annexin V-positive cell lines was increased from 11.29% in the vehicle control group to 22.78 or 42.15% in the TW-37 treatment group (Fig. 1e). Moreover, TW-37-induced apoptosis relied on caspase activation in human oral cancer cell lines (Additional file 1: Figure S2). The findings provided above demonstrated that TW-37 elicits cytotoxicity in human oral cancer cell lines because of apoptosis induced by the caspase-dependent mechanism.

### TW-37 augments apoptosis in human oral cancer cell lines by inhibiting the Mcl-1 expression

**Fig. 2:**
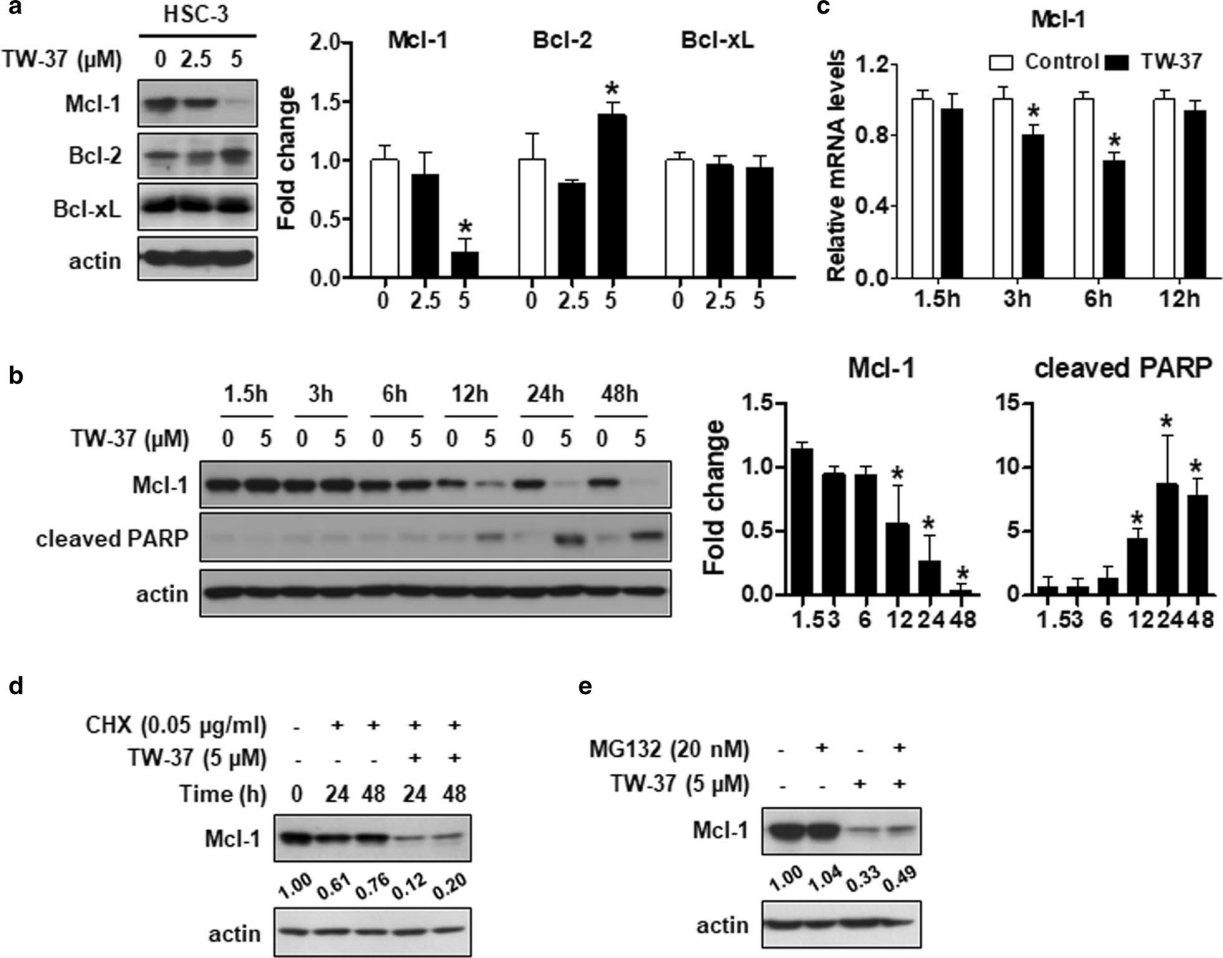
TW-37 induces apoptosis in human oral cancer cell lines by suppressing the Mcl-1 expression. **a** The expressions of myeloid cell leukemia-1 (Mcl-1), Bcl-2, and Bcl-xL were detected using Western blotting. Actin was used as a loading control. **b** The expression levels of Mcl-1 and cleaved PARP were detected using Western blotting. Actin was used as a loading control. **c** Relative mRNA levels of Mcl-1 were measured by qPCR and normalized to GAPDH. All bar graphs represent mean ± SD of three independent experiments. *P < 0.05. **d** HSC-3 cell lines were pretreated with CHX for 1 h, followed by treatment with 5-μM TW-37 for 48 h. The expression of Mcl-1 was analyzed using Western blotting. **e** After treatment with MG132 for 1 h, the HSC-3 cell lines were treated with 5-μM TW-37 for the indicated time points. The protein levels of Mcl-1 were detected with Western blotting. Actin was used as a loading control. Data represent the mean of the two independent experiments

As TW-37 is a potent inhibitor of anti-apoptotic Bcl-2 family proteins, such as Bcl-2, Bcl-xL, and Mcl-1, we assessed whether TW-37-induced apoptosis in human oral cancer cell lines could regulate these proteins. The findings revealed that the Mcl-1 expression notably decreased after the TW-37 treatment, whereas the Bcl-2 and Bcl-xL expressions were inconsistent (Fig. 2a). We observed a decline in the Mcl-1 expression after 12 h TW-37 treatment, and PARP cleavage was coordinately enhanced at similar time points (Fig. 2b). In addition, we performed real-time PCR in a time-dependent manner to determine whether TW-37 could affect the levels of Mcl-1 mRNA. TW-37 markedly decreased the Mcl-1 mRNA levels up to 6 h, alleviated after 12 h, and then weakly decreased after 24 or 48 h (Fig. 2c and Additional file 1: Figure S3). Thus, we next investigated whether TW-37 treatment for 24 or 48 h could regulate the protein stability of Mcl-1 in human oral cancer cell lines. After treatment with CHX to stop new protein synthesis, TW-37 strongly degraded Mcl-1 protein (Fig. 2d). Consistently, the degradation of Mcl-1 protein in cells treated with TW-37 for 24 and 48 h was restored by MG132, a proteasome inhibitor (Fig. 2e). These findings suggested that the suppression of the Mcl-1 expression in a transcriptional and post-translational manner could contribute to TW-37-induced apoptosis in human oral cancer cell lines.

### Ectopic expression of Mcl-1 abolishes TW-37-induced apoptosis in human oral cancer cell lines

**Fig. 3:**
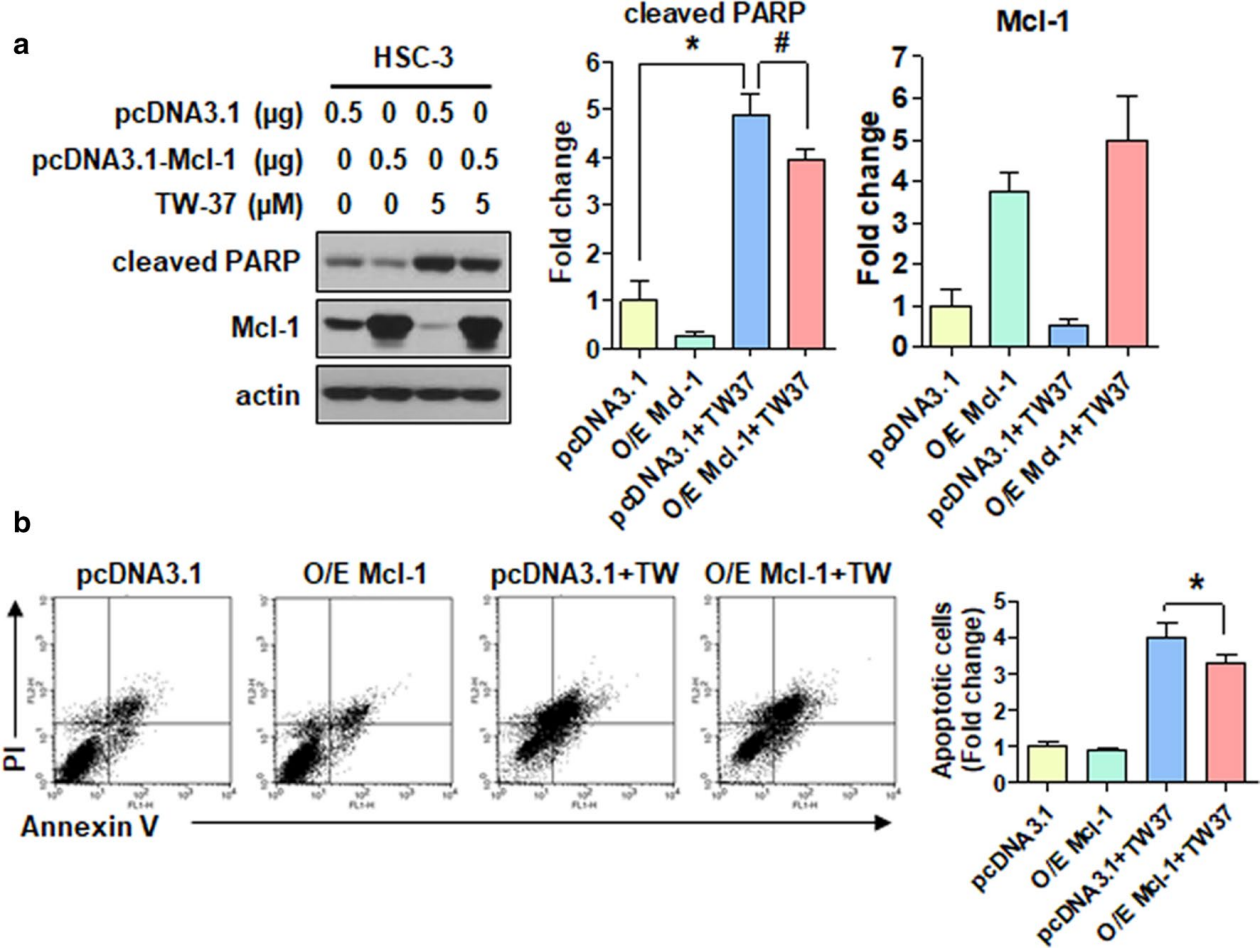
Ectopic expression of Mcl-1 attenuates TW-37-induced apoptosis. HSC-3 cell lines were treated with DMSO or 5-μM TW-37 for 48 h after transiently transfected with 0.5-μg pcDNA3.1 or pcDNA3.1-Mcl-1 for 6 h. **a** Protein levels of myeloid cell leukemia-1 (Mcl-1) and cleaved PARP were determined using Western blotting. Actin was used as a loading control. **b** FACS analysis of annexin V/PI staining. All bar graphs represent mean ± SD of three independent experiments. *P < 0.05; ^#^*P* < 0.05

In this study, cell lines were transiently transfected with pcDNA3.1 (a control vector) and pcDNA3.1-Mcl-1 (to overexpress Mcl-1) to elucidate the biological role of Mcl-1 protein in TW-37-induced apoptosis. The ectopic expression of Mcl-1 modestly attenuated the expression of cleaved PARP after the TW-37 treatment (Fig. 3a). We further established the suppressive effect of Mcl-1 in TW-37-induced apoptosis using flow cytometric analysis. The rate of annexin V-positive cell lines after the TW-37 treatment (52.15%) was partially diminished by the ectopic expression of Mcl-1 (43.02%; Fig. 3b. These findings indicated that the Mcl-1 suppression could be partly needed for TW-37-induced apoptosis in human oral cancer cell lines.

### TW-37 decreases the STAT3 phosphorylation at Tyr^705^ and nuclear translocation in human oral cancer cell lines

**Fig. 4:**
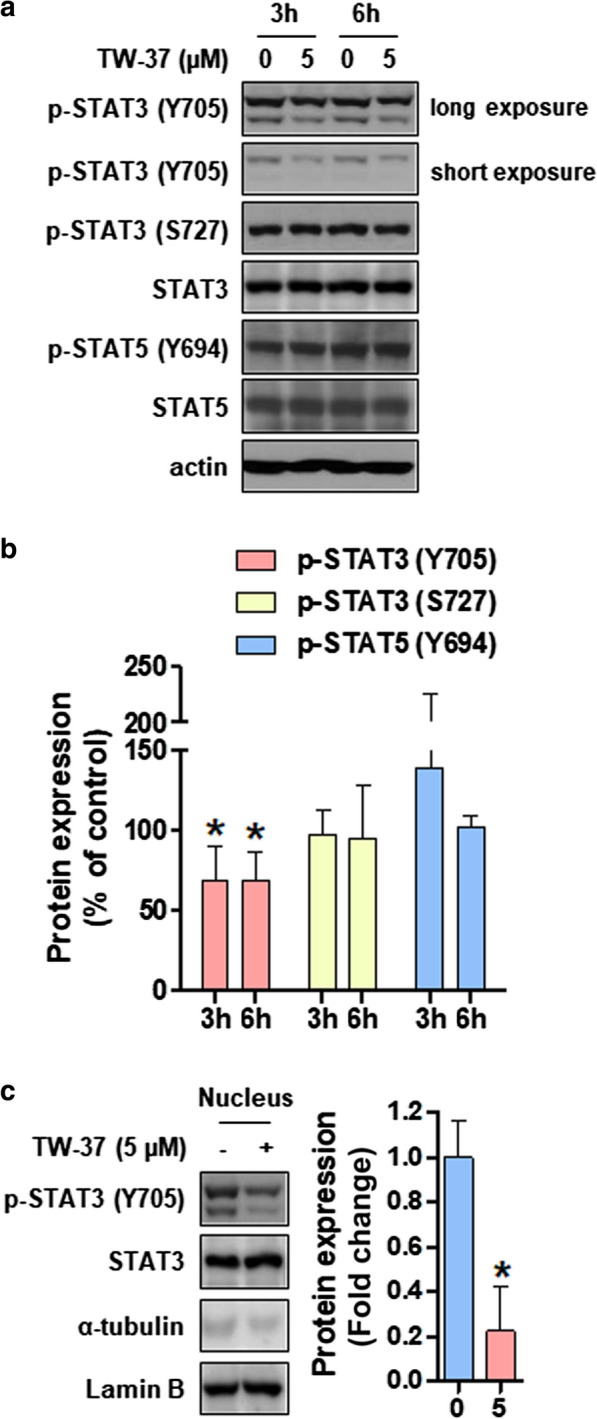
TW-37 inhibits the STAT3 phosphorylation at Tyr^705^ and nuclear translocation. HSC-3 cell lines were treated with DMSO or 5-μM of TW-37 for 3 or 6 h. **a** Protein levels of p-STAT3 (Y705), p-STAT3 (S727), STAT3, p-STAT5 (Y694), and STAT5 were analyzed using Western blotting. Actin was used as a loading control. **b** The bar graphs represent mean ± SD of three independent experiments. *P < 0.05. **c** The expression of nuclear p-STAT3 (Y705) and signal transducer and activator of transcription 3 (STAT3) was detected using Western blotting. In addition, α-tubulin and Lamin B were used as specific markers for cytosolic or nuclear fraction, respectively. The bar graphs represent mean ± SD of three independent experiments. *P < 0.05

STAT signaling pathway plays a vital role in oncogenesis through the modulation of genes involved in anti-apoptotic Bcl-2 proteins such as Mcl-1 [16]. In this study, we explored the phosphorylation of STAT3 and STAT5 at early time points after the TW-37 treatment to determine whether TW-37 could regulate STAT family proteins. TW-37 markedly suppressed the STAT3 phosphorylation at Tyr^705^ in a time-dependent manner but not the STAT3 phosphorylation at Ser^727^ and STAT5 phosphorylation at Tyr^694^ (Fig. 4a, b). Consistently, we observed a reduction of the STAT3 phosphorylation at Tyr^705^ in a concentration-dependent manner in other human oral cancer cell lines, including Ca9.22, HSC-4, and HN22 (Additional file 1: Figure S1d). As the STAT3 phosphorylation exhibits translocation into the nucleus, we examined the phosphorylation status of STAT3 in the nucleus after the TW-37 treatment. Notably, TW-37 inhibited the nuclear translocation of p-STAT3^Tyr705^ (Fig. 4c). These results suggested that TW-37 triggers the suppression of p-STAT3^Tyr705^ and nuclear translocation in human oral cancer cell lines.

### TW-37 enhances the chemosensitivity of cryptotanshinone by modulating the STAT3–Mcl-1 axis in human oral cancer cell lines

**Fig. 5:**
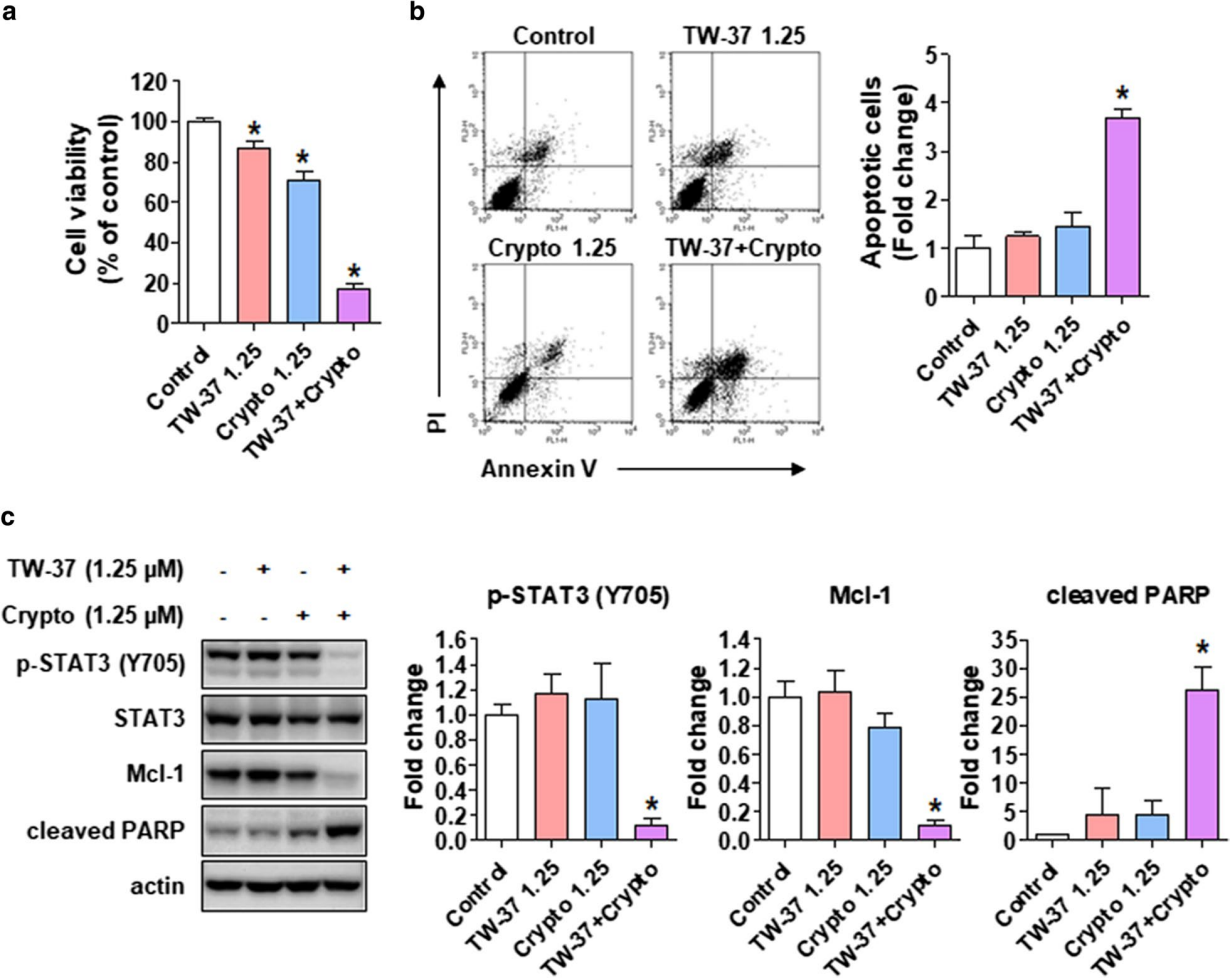
TW-37 augments the chemosensitivity of cryptotanshinone in human oral cancer cell lines by modulating STAT3–Mcl-1 signaling. **a** The cell viability was measured using the trypan blue exclusion assay. **b** FACS analysis of annexin V/PI staining. All bar graphs represent mean ± SD of three independent experiments. **c** Protein levels of p-STAT3 (Y705), signal transducer and activator of transcription 3 (STAT3), and myeloid cell leukemia-1 (Mcl-1) were determined using Western blotting. Actin was used as a loading control. The bar graphs represent mean ± SD of three independent experiments. *P < 0.05

We optimized the concentrations of TW-37 and cryptotanshinone to exclude their direct cytotoxic efficiency on human oral cancer cell lines to investigate the synergistic effect of TW-37 with cryptotanshinone as a potent STAT3 inhibitor. The combined treatment of TW-37 and cryptotanshinone potently decreased the growth of HSC-3 cell lines compared with either TW-37 or cryptotanshinone alone (Fig. 5a). Moreover, the rate of annexin V-positive cell lines was enhanced after the combined treatment of TW-37 and cryptotanshinone (Fig. 5b). Furthermore, we confirmed the levels of p-STAT3 ^Tyr705^ and Mcl-1 by Western blotting to elucidate whether the synergistic effect of TW-37 with cryptotanshinone correlated with STAT3–Mcl-1 signaling. The combined treatment of TW-37 and cryptotanshinone remarkably abolished the expression of p-STAT3 ^Tyr705^ and Mcl-1 compared with either TW-37 or cryptotanshinone alone, resulting in a potent induction of apoptosis (Fig. 5c). These findings suggested that TW-37 can synergistically induce the chemosensitivity of cryptotanshinone by inducing apoptosis through suppressing STAT3–Mcl-1 signaling in human oral cancer cell lines.

## Discussion

Although targeting anti-apoptotic Bcl-2 family proteins is a promising and well-established therapeutic strategy for the treatment of various tumors, developing Bcl-2 inhibitors and exploring their efficiency because of the oncogenic potential of anti-apoptotic Bcl-2 family proteins are still worthwhile. This study reports the single and chemosensitizing effects of TW-37, a potent inhibitor of anti-apoptotic Bcl-2 family proteins, for the treatment of oral cancer. Our analysis of the effect of TW-37 on anti-apoptotic Bcl-2 family proteins in human oral cancer cell lines revealed that TW-37 suppressed the Mcl-1 expression at the transcriptional and post-translational levels, resulting in apoptosis (Fig. 2). Mcl-1, an anti-apoptotic member of Bcl-2 family proteins, is regulated at the transcriptional, post-transcriptional, and post-translational levels upon several factors such as growth factors [17]. In addition, Mcl-1 is overexpressed in various types of cancer, which is related to the evasion of cell death and acquisition of resistance to chemotherapeutic drugs [18]. Specifically, we previously established that the Mcl-1 expression was higher in human oral tumors compared with healthy oral mucosa and was accountable for neoplastic cell transformation under 12-O-tetradecanoylphorbol-13-acetate or epidermal growth factor [19]. This study reported that the ectopic expression of Mcl-1 partly declined the apoptotic activity of TW-37 in human oral cancer cell lines, suggesting that regulation of the Mcl-1 expression is needed for TW-37-induced apoptosis in human oral cancer cell lines (Fig. 3). Hence, seeking a Mcl-1-targeting inhibitor could provide a potential therapeutic benefit for oral cancer therapy.

STAT3, one of the STAT family members, is a transcription factor playing a vital role in pathological processes of cancer. Notably, the constitutive phosphorylation of STAT3 correlates with tumor initiation and progression by suppressing apoptosis and inducing proliferation, invasion, metastasis, and angiogenesis, which is accompanied by dimerization, translocation to the nucleus, and binding to promoters of target genes [20, 21]. We previously illustrated that the phosphorylated STAT3 levels markedly elevated in tissues obtained from patients with oral cancer compared with tissues obtained from the healthy oral mucosa, suggesting that p-STAT3 is a good prognostic indicator of oral cancer [22]. In addition, Zhou et al. demonstrated that the deletion of putative STAT3 binding site within the promoter of Mcl-1 abolished the promoter activity of Mcl-1 in lung cancer cell lines, suggesting the transcriptional regulation of Mcl-1 by STAT3 [15]. This study established that the TW-37 treatment markedly abrogated the p-STAT3^Tyr705^ levels in human oral cancer cell lines, thereby inhibiting the nuclear translocation of p-STAT3^Tyr705^ (Fig. 4). Using STAT3 inhibitors such as cryptotanshinone and stattic, we also investigated whether STAT3 signaling affects the Mcl-1 level; this was performed to support the evidence that p-STAT3 is the upstream signal of Mcl-1. The results showed that these STAT3 inhibitors significantly decreased the cell viability and induced apoptosis in human oral cancer cell lines via the downregulation of Mcl-1 (Additional file 1: Figure S4a–c). In contrast, the ectopic expression of Mcl-1 had no effect on the activation of STAT3 (Additional file 1: Figure S4d). Moreover, we compared the effects of TW-37 with those of ABT-737 on p-STAT3-Mcl-1 signaling; TW-37 significantly inhibited p-STAT3-Mcl-1 signaling; however, ABT-737 did not affect it although both inhibitors showed similar apoptotic efficiency (Additional file 1: Figure S5). Thus, our findings indicate that the decreased expression of Mcl-1 protein induced by TW-37 in human oral cancer cell lines could arise from the inhibition of STAT3 activation and nuclear translocation, thereby validating H1 of this study. Nevertheless, further studies are required to demonstrate the role of STAT3 on reduced Mcl-1 promoter activity upon TW-37 treatment.

To date, numerous STAT3-targeted therapies inhibiting the SH2 domain, upstream tyrosine kinase, and DNA-binding domain have been considered potential therapeutic approaches for clinical applications because targeting STAT3 could be a reasonable strategy for the development of chemotherapeutic drugs against cancer [23]. Cryptotanshinone, one of the major active ingredients isolated from the traditional Chinese medicinal herb *Salvia miltiorrhiza* Bunge (red sage), is a STAT3 inhibitor that binds to the SH2 domain of STAT3 [24]. In addition, cryptotanshinone reportedly suppressed the cell proliferation and induced cell cycle arrest and apoptosis in various cancer types by suppressing JAK2–STAT3 signaling, as well as PI3K-Akt-NF-κB signaling [25–27]. Reportedly, cryptotanshinone enhances the chemosensitivity of conventional chemotherapeutic drugs, such as doxorubicin and paclitaxel, in cancer cell lines by inhibiting the STAT3 signaling pathway [28, 29]. Accordingly, we optimized the concentrations of TW-37 and cryptotanshinone to exclude their direct cytotoxic efficiency on human oral cancer cell lines. Our findings revealed that the combination of TW-37 and cryptotanshinone further enhanced the chemosensitivity of human oral cancer cell lines than a single drug alone, which was, perhaps, caused by the inhibition of STAT3–Mcl-1 signaling (Fig. 5), thereby validating H2 of this study. Hence, this study suggests that TW-37 potentiates the apoptotic effect of cryptotanshinone in human oral cancer cell lines by suppressing STAT3–Mcl-1 signaling.

## Conclusion

This study demonstrates that TW-37 alone and in combination with cryptotanshinone exerts a potent apoptotic effect on human oral cancer cell lines by inhibiting STAT3–Mcl-1 signaling. Overall, this study highlights that targeting STAT3 using TW-37 could be an attractive approach in enhancing the prognosis of patients with oral cancer in the future.

## Supplementary information

**Additional file 1: Figure S1.** TW-37 induces apoptosis in human oral cancer cell lines by suppressing p-STAT3Tyr705. Ca9.22, HSC-4, and HN22 cell lines were treated with 2.5- and 5-μM TW-37 for 48 h. a The cell viability was measured using the trypan blue exclusion assay. Bar graphs represent the mean ± SD values of three independent experiments. *P < 0.05. b Protein levels of cleaved caspase-3 and cleaved PARP were analyzed using Western blotting. Actin was used as a loading control. c All cell lines stained with DAPI were visualized by fluorescence microscopy (magnification, ×400). d The expressions of p-STAT3 (Y705) and STAT3 were detected using Western blotting. Actin was used as a loading control.** Figure S2.** TW-37-induced apoptosis depending on caspase activation in human oral cancer cell lines. HSC-3 cell lines were pretreated with 10-μM Z-VAD for 2 h with/without 5-μM TW-37 for 48 h. a Protein levels of cleaved PARP were analyzed using Western blotting analysis. Actin was used as a loading control. b Bar graphs represent the mean ± SD values of three independent experiments. *P < 0.05; #P < 0.05.** Figure S3.** TW-37 slightly decreases Mcl-1 mRNA levels at 24 and 48 h. The HSC-3 cell lines were treated with 5-μM of TW-37 for 24 or 48 h. Relative mRNA levels of Mcl-1 were measured with qPCR and normalized to GAPDH. Bar graphs represent the mean ± SD values of triplicate experiments. *P < 0.05.** Figure S4.** STAT3 inactivation is sufficient to induce apoptosis via the inhibition of Mcl-1 expression. The HSC-3 cell lines were treated with 5-μM of cryptotanshinone or stattic for 24 h. a The cell viability was measured using the trypan blue exclusion assay. b Protein levels of p-STAT3 (Y705), STAT3, Mcl-1, and cleaved PARP were analyzed using Western blotting analysis. Actin was used as a loading control. c Relative mRNA levels of Mcl-1 were measured with qPCR and normalized to GAPDH. d The HSC-3 cell lines were transiently transfected with 0.5-μg pcDNA3.1 or pcDNA3.1-Mcl-1 for 6 h. Protein levels of p-STAT3 (Y705) and STAT3 were determined using Western blotting analysis. Actin was used as a loading control. All bar graphs represent the mean ± SD values of three independent experiments. *P < 0.05.** Figure S5.** TW-37-induced apoptosis is mediated by the suppression of STAT3-Mcl-1 signaling unlike ABT-737. The HSC-3 cell lines were treated with the indicated concentrations of TW-37 or ABT-737 for 48 h. a Protein levels of p-STAT3 (Y705), STAT3, Mcl-1, and cleaved PARP were analyzed using Western blotting analysis. Actin was used as a loading control. b Bar graphs represent the mean ± SD values of three independent experiments. *P < 0.05.

## Data Availability

The datasets used and/or analyzed during the current study are available from the corresponding author upon reasonable request.
