# Supplementary material for: Cryptotanshinone chemosensitivity potentiation by TW-37 in human oral cancer cell lines by targeting STAT3–Mcl-1 signaling
Source: Cancer Cell Int. 2020 Aug 26;20:405. doi: 10.1186/s12935-020-01495-2 (PMC7448991; doi:10.1186/s12935-020-01495-2)
Supplement: Supplementary file 1 — Additional file 1: Figure S1. TW-37 induces apoptosis in human oral cancer cell lines by suppressing p-STAT3Tyr705. Ca9.22, HSC-4, and HN22 cell lines were treated with 2.5- and 5-μM TW-37 for 48 h. a The cell viability was measured using the trypan blue exclusion assay. Bar graphs represent the mean ± SD values of three independent experiments. *P < 0.05. b Protein levels of cleaved caspase-3 and cleaved PARP were analyzed using Western blotting. Actin was used as a loading control. c All cell lines stained with DAPI were visualized by fluorescence microscopy (magnification, ×400). d The expressions of p-STAT3 (Y705) and STAT3 were detected using Western blotting. Actin was used as a loading control. Figure S2. TW-37-induced apoptosis depending on caspase activation in human oral cancer cell lines. HSC-3 cell lines were pretreated with 10-μM Z-VAD for 2 h with/without 5-μM TW-37 for 48 h. a Protein levels of cleaved PARP were analyzed using Western blotting analysis. Actin was used as a loading control. b Bar graphs represent the mean ± SD values of three independent experiments. *P < 0.05; #P < 0.05. Figure S3. TW-37 slightly decreases Mcl-1 mRNA levels at 24 and 48 h. The HSC-3 cell lines were treated with 5-μM of TW-37 for 24 or 48 h. Relative mRNA levels of Mcl-1 were measured with qPCR and normalized to GAPDH. Bar graphs represent the mean ± SD values of triplicate experiments. *P < 0.05. Figure S4. STAT3 inactivation is sufficient to induce apoptosis via the inhibition of Mcl-1 expression. The HSC-3 cell lines were treated with 5-μM of cryptotanshinone or stattic for 24 h. a The cell viability was measured using the trypan blue exclusion assay. b Protein levels of p-STAT3 (Y705), STAT3, Mcl-1, and cleaved PARP were analyzed using Western blotting analysis. Actin was used as a loading control. c Relative mRNA levels of Mcl-1 were measured with qPCR and normalized to GAPDH. d The HSC-3 cell lines were transiently transfected with 0.5-μg pcDNA3.1 or pcDNA3.1-Mcl [file 12935_2020_1495_MOESM1_ESM.docx]

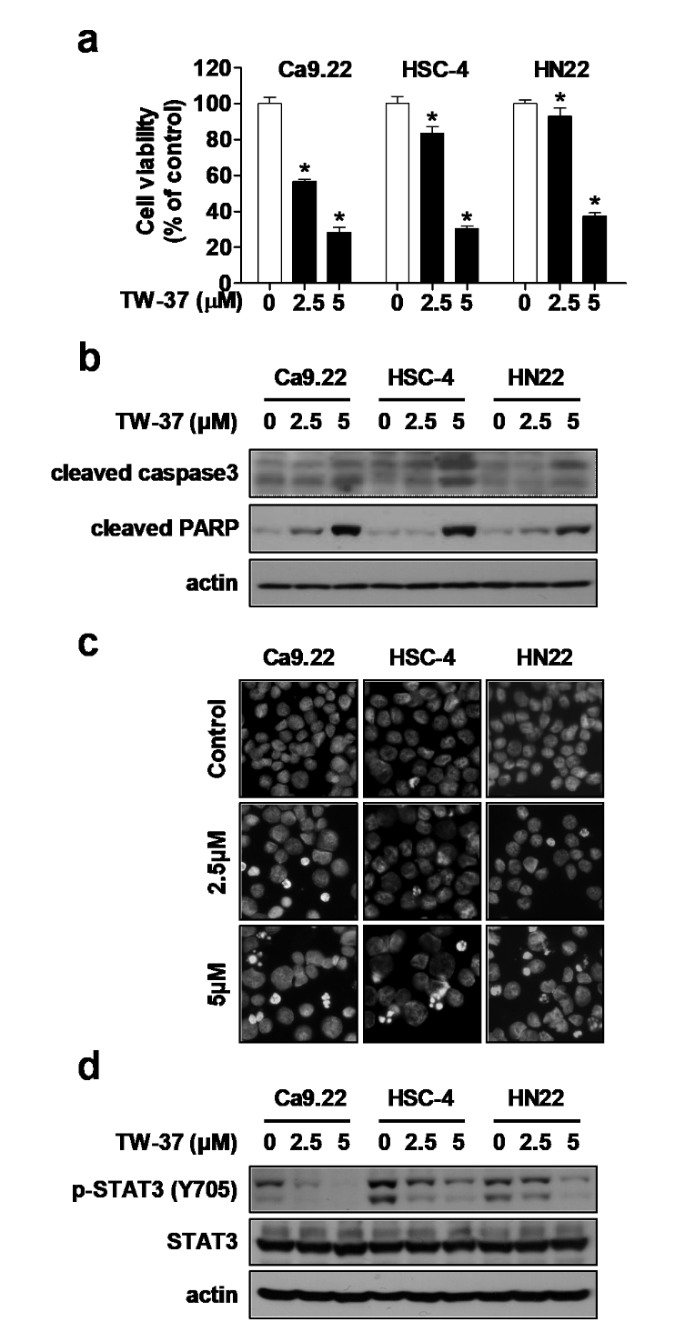


**Fig. S1.** TW-37 induces apoptosis in human oral cancer cell lines by suppressing p-STAT3^Tyr705^. Ca9.22, HSC-4, and HN22 cell lines were treated with 2.5- and 5-μM TW-37 for 48 h. **a** The cell viability was measured using the trypan blue exclusion assay. Bar graphs represent the mean ± SD values of three independent experiments. ^*^*P* < 0.05. **b** Protein levels of cleaved caspase-3 and cleaved PARP were analyzed using Western blotting. Actin was used as a loading control. **c** All cell lines stained with DAPI were visualized by fluorescence microscopy (magnification, ×400). **d** The expressions of p-STAT3 (Y705) and STAT3 were detected using Western blotting. Actin was used as a loading control.

**
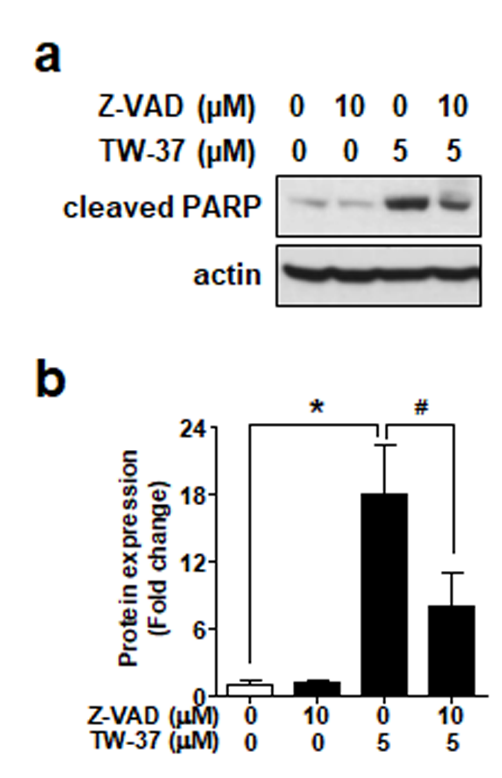
**

**Fig. S2.** TW-37-induced apoptosis depending on caspase activation in human oral cancer cell lines. HSC-3 cell lines were pretreated with 10-μM Z-VAD for 2 h with/without 5-μM TW-37 for 48 h. **a** Protein levels of cleaved PARP were analyzed using Western blotting analysis. Actin was used as a loading control. **b** Bar graphs represent the mean ± SD values of three independent experiments. ^*^*P* < 0.05; ^#^*P* < 0.05.

**
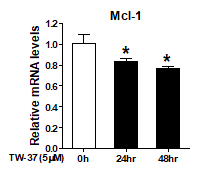
**

**Fig. S3.** TW-37 slightly decreases Mcl-1 mRNA levels at 24 and 48 h. The HSC-3 cell lines were treated with 5-μM of TW-37 for 24 or 48 h. Relative mRNA levels of Mcl-1 were measured with qPCR and normalized to GAPDH. Bar graphs represent the mean ± SD values of triplicate experiments. ^*^*P* < 0.05.

**
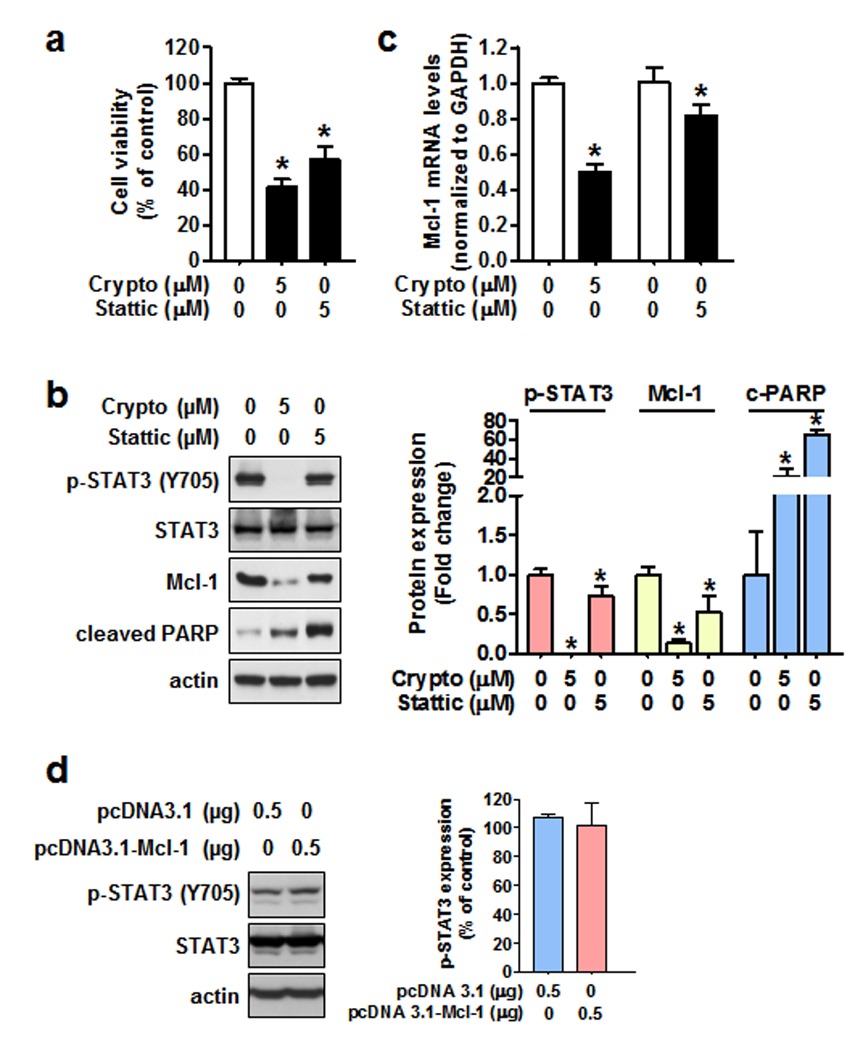
**

**Fig. S4.** STAT3 inactivation is sufficient to induce apoptosis via the inhibition of Mcl-1 expression. The HSC-3 cell lines were treated with 5-μM of cryptotanshinone or stattic for 24 h. **a** The cell viability was measured using the trypan blue exclusion assay. **b** Protein levels of p-STAT3 (Y705), STAT3, Mcl-1, and cleaved PARP were analyzed using Western blotting analysis. Actin was used as a loading control. **c** Relative mRNA levels of Mcl-1 were measured with qPCR and normalized to GAPDH. **d** The HSC-3 cell lines were transiently transfected with 0.5-μg pcDNA3.1 or pcDNA3.1-Mcl-1 for 6 h. Protein levels of p-STAT3 (Y705) and STAT3 were determined using Western blotting analysis. Actin was used as a loading control. All bar graphs represent the mean ± SD values of three independent experiments. ^*^*P* < 0.05.


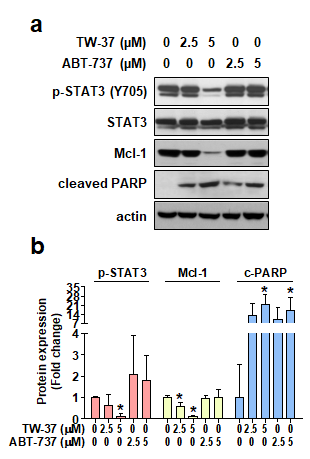


**Fig. S5.** TW-37-induced apoptosis is mediated by the suppression of STAT3-Mcl-1 signaling unlike ABT-737. The HSC-3 cell lines were treated with the indicated concentrations of TW-37 or ABT-737 for 48 h. **a** Protein levels of p-STAT3 (Y705), STAT3, Mcl-1, and cleaved PARP were analyzed using Western blotting analysis. Actin was used as a loading control. **b** Bar graphs represent the mean ± SD values of three independent experiments. ^*^*P* < 0.05.
